# Congenital Haemolytic Jaundice in the Adult

**Published:** 1917-09

**Authors:** A. Chauffard

**Affiliations:** Faculty of Medicine of Paris


### CONGENITAL HAEMOLYTIC JAUNDICE IN THE ADULT.

By Professor A. Chauffard,

Of the Faculty of Medicine of Paris

The first appearance in scientific circles of haemolytic jaundice in the adult was in the form of the family hereditary variety. This definition is based on the first case published by Minkowsky in 1900. It was that of a family in which eight cases of jaundice were observed in three succeeding generations. The disease, says Minkowsky, is characterized by chronic jaundice with urobilinuria, splenomegaly, and renal siderosis.

My own case was that of a medical student who had suffered from well-marked jaundice from birth, and he was a golden yellow. At first I wondered whether he had a congenital mal-formation of the biliary apparatus calling for surgical intervention, but laboratory research showed remarkable fragility of the blood corpuscle which is the starting point of the haemolytic theory of certain cases of jaundice. Since that date many such cases have been recorded.

One of the most recent was that of a lad, twenty years of age, who sought admission on account of his yellow color and occasional attacks of pain in the left hypochondrium. He says he has had this pain off and on ever since the age of five. He has also suffered from intestinal disturbances with alternations of constipation and diarrhoea, leading his medical advisers to suspect tuberculous peritonitis on the occasion of an acute attack. He was able, however, to go back to work with merely slight pain in the hypochondrium and left shoulder. He asserts that the motions were never clay colored, but always of a dark greenish-bronze.

In 1910, consequent upon a journey by rail, he was seized with severe pain in the hypochondrium. He presented a moderate cutaneo-mucous jaundice of a greyish anaemic tint on the skin, golden yellow in the conjunctivae. The urine was clear and did not give Gmelin's reaction, though it contained a notable proportion of urobilin. The liver was about normal, vertical dulness 12 cm. Examination of the blood showed unquestionable cholaemia.

It is seen, therefore, that there is a chronic congenital infantile form of jaundice. In most instances the jaundice is the initial symptom and the patients are yellow from the start. In this case the father tells us that the child was as yellow as a guinea when he came into the world. In other cases the jaundice is secondary to an initial phase of icteric anaemia.

The patients are first pale, then become yellow. Urobilin is invariably found in the blood, because urobilinaemia accompanies urobilinuria.

Another important feature of this diseaes is the non-decoloration of the faeces. This is a detail which must always be enquired into when there is any reason to suspect haemolytic jaundice. In these cases the stools are not clayey and pasty, as in retention jaundice. On the contrary, they are over-colored, greenish, even spinach green. Then, too, we cannot but be struck by the fact that these patients maintain a very decent standard of health in spite of the jaundice, which, for that matter, is not invariably present.

Such patients turn yellow or cease to be yellow according to circumstances. Diet does not count for much, but they are neuropathic subjects in whom emotions are apt to increase the jaundice for a time.

Congenital jaundice is also characterized by the three following negative signs, viz., absence of the itching, which is such a distressing symptom in other icteric patients, absence of brady-cardia and xanthelasma.

The production of palpebral xanthelasma is constant in cases of chronic protracted jaundice. Its absence in conginital icterics is to be explained by the biological constitution of this little tumor, as shown by Laroche and myself. In a monograph on the subject we established the fact the xanthelasma is a local affection due to the deposit of cholesterin in the dermis. Its formation is therefore in relationship with the increase of cholesterin in the blood, and it is only met with in patients with jaundice associated with hypercholesteraemia.

On the other hand, this congenital jaundice, from the very fact that it is not due to retention, is not accompanied by decoloration of the stools. On the contrary, the pigment is in excess constituting what Stedelmann called peiochromic jaundice.

The spleen as a rule measures from fifteen to twenty centimetres. It is best seen by the aid of a radioscopy.

These patients are often rather delicate and have a pallid hue, though in reality more icteric than anaemic. This is in contrast with the victims of acquired haemolytic jaundice who are often more anaemic than icteric.

A series of complications may determine grades passing from mild cases to comparatively serious cases.

Clinical examination already affords us fairly strong presumptions as to the existence of congenital haemolytic jaundice, but we must never remain satisfied with merely clinical evidence, and certainly can only be provided by laboratory investigation.

Under normal circumstances the blood is maintained in a state of isotonic equilibrium by means of erythropoiesis. Now it sometimes happens that the red corpuscles present what is known as globular fragility. In such case erythropoiesis is exaggerated and the bile pigments derived from the haemoglobin pass into the circulation. This leads to non-compensated forms of haemolytic jaundice, to the pernicious icterogenous anaemia described by Laroche and myself. The compensated form corresponds to congenital haemolytic jaundice.

We ascertain the existence of globular fragility by the method introduced by Vaquez and Riviere. Roctor Rendu always found a very pronounced diminution of globular resistance in these cases of congenital jaundice. Or we may have recourse to Widal's method of deplasmatized blood, which is more sensitive than the preceding and may be found useful in certain cases.

Haemolytic jaundice then is characterized by a diminution of globular resistance. In the second place it is associated with a reduction of the average diameter of the red corpuscles and anysocytosis, i.e., inequality and deformation of the corpuscles. In this patient the average diameter is from three to five microns. In jaundice, by retention, on the contrary, we get increased resistance and diameter, so that there is absolute opposition between the two types.

Haemolytic jaundice is also peculiar by reason of the biological feature of the presence of a large number of granular red corpuscles, which are shown to be present by means of Pappenheim's reactive.

These granular red corpuscles must not be looked upon as changed red corpuscles, but in reality as elements of abnormal, atypical, pathological regeneration. I have convinced myself that this is the correct explanation by injecting eel serum into animals, which, as you know, has for effect to rapidly destroy the red corpuscles. Now it was not at the time that this destruction takes place that we find granular corpuscles in the blood of the subjects, but only when regeneration is beginning to take place.

The absence of auto-agglutination and of auto-haemolysis completes the haemotological characters of congenital haemolytic icterus.—Medical Press and Circular.

